# Identifying Inorganic Turbidity in Water Samples as Potential Loss Factor During Nucleic Acid Extraction: Implications for Molecular Fecal Pollution Diagnostics and Source Tracking

**DOI:** 10.3389/fmicb.2021.660566

**Published:** 2021-10-20

**Authors:** Rita B. Linke, Sibel Zeki, René Mayer, Katharina Keiblinger, Domenico Savio, Alexander K. T. Kirschner, Georg H. Reischer, Robert L. Mach, Regina Sommer, Andreas H. Farnleitner

**Affiliations:** ^1^Research Group Environmental Microbiology and Molecular Diagnostics 166/5/3, Institute of Chemical, Environmental and Bioscience Engineering, TU Wien, Vienna, Austria; ^2^Department of Marine Environment, Institute of Marine Sciences and Management, Istanbul University, Istanbul, Turkey; ^3^Department of Forest and Soil Sciences, Institute of Soil Research, University of Natural Resources and Life Sciences Vienna, Vienna, Austria; ^4^Division Water Quality and Health, Department Pharmacology, Physiology and Microbiology, Karl Landsteiner University of Health Sciences, Krems an der Donau, Austria; ^5^Institute for Hygiene and Applied Immunology, Medical University of Vienna, Vienna, Austria; ^6^Research Area Molecular Diagnostics, Department IFA-Tulln, Institute of Chemical, Environmental and Bioscience Engineering, TU Wien, Tulln, Austria; ^7^Research Division Biochemical Technology, Institute of Chemical, Environmental and Bioscience Engineering, TU Wien, Vienna, Austria; ^8^Unit of Water Microbiology, Institute for Hygiene and Applied Immunology, Medical University of Vienna, Vienna, Austria

**Keywords:** water quality, inorganic turbidity, DNA extraction, sample process control, microbial source tracking, fecal pollution diagnostics

## Abstract

Molecular diagnostic methods are increasingly applied for food and environmental analysis. Since several steps are involved in sample processing which can affect the outcome (e.g., adhesion of DNA to the sample matrix, inefficient precipitation of DNA, pipetting errors and (partial) loss of the DNA pellet during DNA isolation), quality control is essential at all processing levels. In soil microbiology, particular attention has been paid to the inorganic component of the sample matrix affecting DNA extractability. In water quality testing, however, this aspect has mostly been neglected so far, although it is conceivable that these mechanisms have a similar impact. The present study was therefore dedicated to investigate possible matrix effects on results of water quality analysis. Field testing in an aquatic environment with pronounced chemo-physical gradients [total suspended solids (TSS), inorganic turbidity, total organic carbon (TOC), and conductivity] indicated a negative association between DNA extractability (using a standard phenol/chloroform extraction procedure) and turbidity (spearman ρ = −0.72, *p* < 0.001, *n* = 21). Further detailed laboratory experiments on sediment suspensions confirmed the hypothesis of inorganic turbidity being the main driver for reduced DNA extractability. The observed effects, as known from soil samples, were also indicated to result from competitive effects for free charges on clay minerals, leading to adsorption of DNA to these inorganic particles. A protocol modification by supplementing the extraction buffer with salmon sperm DNA, to coat charged surfaces prior to cell lysis, was then applied on environmental water samples and compared to the standard protocol. At sites characterized by high inorganic turbidity, DNA extractability was significantly improved or made possible in the first place by applying the adapted protocol. This became apparent from intestinal enterococci and microbial source tracking (MST)-marker levels measured by quantitative polymerase chain reaction (qPCR) (100 to 10,000-fold median increase in target concentrations). The present study emphasizes the need to consider inorganic turbidity as a potential loss factor in DNA extraction from water-matrices. Negligence of these effects can lead to a massive bias, by up to several orders of magnitude, in the results of molecular MST and fecal pollution diagnostics.

## Introduction

Methods for molecular fecal pollution diagnostics and microbial source tracking (MST) to quantify fecal pollution and allocate it to distinct pollution sources, are increasingly used worldwide (e.g., Wuertz et al., 2011; Reischer et al., 2013; Odagiri et al., 2015; Boehm et al., 2016; Mayer et al., 2018). Usually, these parameters are determined by applying a quantitative polymerase chain reaction (qPCR) to DNA extracted from environmental samples (e.g., water, soil, sediment). A variety of qPCR-based methods are suited for molecular diagnostics of fecal pollution and MST applications as well as the detection of microbial indicators and pathogens for monitoring water quality due to their achievable precision, specificity, and sensitivity. Yet, for the application to environmental samples, a number of possible sources of error have to be considered. In particular, it is the quantity and quality of extracted DNA that might be affected by the sample matrix or the co-extraction of PCR inhibiting substances (Green and Field, 2012; Hill et al., 2015; Lever et al., 2015; Rocha and Manaia, 2020). Moreover, genetic markers (e.g., for fecal indication, pathogenic microbes) are typically present at low to very low concentrations which is why they, in many cases, have to be enriched (filtration) or purified during sample processing. Therefore, the analysis of microbial samples remains a practical and technological challenge when it comes to confidence in the measurements, especially for measurements made at the point of interest or point of care where results are used to inform critical decision-making (Da Silva et al., 2016).

In contrast to molecular investigations of microbiological water quality, the aspect of the sample matrix influencing DNA extraction has attracted much attention in studies on soil microbiology (e.g., Herrera and Cockell, 2007; Saeki et al., 2010; Paulin et al., 2013; Lever et al., 2015). From these studies, it is known that the performance of DNA extraction can be directly related to the soil’s composition (Frostegard et al., 1999; Miller et al., 1999; Lombard et al., 2011; Paulin et al., 2013). Among the various soil types studied it was shown that mainly those with a high clay content (Cai et al., 2006; Yu et al., 2013; Lever et al., 2015; Engel et al., 2019) are likely to adsorb and bind DNA. Taking into consideration that many rivers and lakes transport large quantities of fine sediment and clay minerals, an effect on DNA extraction efficiency is highly conceivable. In particular, under increased discharge conditions during flooding or in the presence of strong wind sediments are mobilized resulting in high inorganic turbidity, possibly interfering with DNA extraction efficiency in samples taken under those conditions. Also, the increased occurrence of heavy rainfall events as a result of climate change in combination with deforestation, clearance, and associated increased soil erosion can lead to an increased input of sediment into water bodies and temporarily high turbidity. Despite these aspects however, this issue for water quality analysis has only recently been the subject of first studies (Lebuhn et al., 2004; Haugland et al., 2012; Shanks et al., 2016; Li et al., 2019; Rocha and Manaia, 2020).

The present study aimed at testing the hypothesis that inorganic turbidity, if present, could affect the efficiency of DNA extraction, and, as a consequence, the reliability and reproducibility of quantitative molecular methods applied for water quality testing. The test strategy comprised three levels: (i) evaluation of the postulated detrimental effect on environmental samples from an aquatic habitat showing a strong gradient of chemo-physical parameters (inorganic turbidity, organic matter content, and salinity), (ii) experimental testing of the hypothesis in the laboratory on samples specifically generated to simulate an extreme gradient of inorganic turbidity and trying to understand the type of effects by applying competitive antagonists, and finally, (iii) adapting the routinely used DNA extraction protocol based on the formerly obtained findings and applying it on different environmental samples from the study area. As study area, a shallow steppe lake and a soda lake were chosen in order to cover a broad range of different sample matrices. A sample process control, employed on a sample-to-sample basis, was used to determine DNA extraction efficiency.

## Materials and Methods

### Model Study Area

The study area was located in National Park Lake Neusiedl, which is situated in the lowlands of Eastern Austria. The area included a large steppe lake (Lake Neusiedl) as well as several smaller lakes. These habitats are known for both high and fluctuating turbidity values which is why this area was chosen for the present study. Lake Neusiedl is surrounded by a reed belt, which is characterized by lower turbidity but higher contents of organic material. Therefore, this area in its complexity, offered a wide variety of different aquatic habitats according to their chemo-physical parameters [inorganic and organic turbidity, total suspended solids (TSS), total organic carbon (TOC), and conductivity] and water matrices making it ideally suited to test the hypothesis of the sample matrix interfering with sample processing efficiency. Samples were taken at six sampling sites: soda lake Oberer Stinker (OS), Lake Neusiedl (L), reed belt of Lake Neusiedl (RB), wastewater stabilization pond (P), un-treated (W-ut), and treated wastewater (W-t). The latter two were included in the study due to the high content of organic matter.

Lake Neusiedl (L) is a shallow alkaline brown-water steppe lake which is a nearly closed system without natural outlet, receiving 80% of its water input from precipitation, 20% from rivers and losing about 90% by evapotranspiration and 10% by an artificial channel regulated by a sluice gate (Kirschner et al., 2008; Soja et al., 2014). More than half (56%) of the lake area (320 km^2^) is a covered by a *Phragmites australis*-stand (Hietz, 1992). Within the reed cover, water is not turbid due to reduced wind exposure and brown in color due to humic substances (Dokulil and Herzig, 2009). It is due to the extreme shallowness of the lake (mean depth 1.1 m, maximum depth 2.0 m) and wind exposure, that the lake is characterized by high concentrations of suspended solids (up to 800 mg dw L^–1^) and large annual variations in temperature (28°C in summer and 1°C in winter) (Wolfram, 1996; Dokulil and Herzig, 2009). Chemically, the lake is characterized by elevated levels of alkalinity (5.0–14.6 meq L^–1^), conductivity (1,100–3,100 μS) and pH (8.3–8.9) in comparison to typical freshwater lakes, with considerable spatial and temporal variability (Wolfram, 2006; Schauer et al., 2015).

The hypertrophic shallow soda lake (Oberer Stinker, OS) is characterized by high total salt concentrations and turbidity (Eiler et al., 2003; Kirschner et al., 2004). It was formed by mineral solutes ascending with the groundwater flux (Krachler et al., 2000) and is characterized by pH values ranging from 9.4 to 10 (Eiler et al., 2003). Na^+^ is the dominating cation, and HCO_3_^–^, CO_3_^2–^, Cl^–^, and SO_4_^2–^ represent the major anions. Salinity of the soda lake varies strongly with seasons (Eiler et al., 2003).

### Sampling for Test Series I—Evaluating Matrix Effects in Field Samples

Water and wastewater samples were collected over a period of 5 months in sterile 1 L sampling bottles (Nalgene, United Kingdom), stored in the dark in cooling boxes at 4°C during transport, and processed within 6 h after collection. A given volume of water (surface water: 100 mL, un-treated waste water: 10 mL, treated waste water: 50 mL) was filtered through Isopore 0.2 μm polycarbonate membrane filters (Millipore, Bedford, MA, United States). Immediately after filtration, the filters were frozen and stored at −80°C until nucleic acid extraction. Six independent filtrations were done for each sample. One replicate was used as an un-spiked control. The other five replicate samples were directly spiked with 5 × 10^7^ cells of the defined target cell standard (DeTaCS; see section “Sample Process Control”) during filtration. On each sampling occasion, an additional unused filter was placed directly into a 1.5 mL extraction vial as a blank filter control.

### Sampling for Test Series II—Simulating an Extreme Gradient of Suspended Solids

To prepare samples mimicking an extreme gradient of suspended solid content, 20 L of water from Lake Neusiedl were sampled in sterile plastic sampling bottles (Nalgene, United Kingdom). In addition, a sediment sample from the bottom of the lake was taken by carefully dragging a sterile glass bottle (Duran Group, Germany) over the ground of the lake collecting the uppermost 2–3 cm of deposited sediment. Water and sediment samples were stored in the dark in cooling boxes at 4°C and were transported to the laboratory. Samples were stored over night at 4°C to ensure settling of the fine sediment fraction and were then processed. To evaluate the influence of suspended solids on DNA extractability, sediment-enriched samples containing 9–220 mg sediment L^–1^ water were prepared in the laboratory by suspending lake bottom sediment in water from Lake Neusiedl (for details regarding the production of sediment-enriched samples see Supplementary Data 1). From samples containing 9, 34, and 106 mg sediment L^–1^ water, 10 independent filtrations were performed which were used for further testing to modify and adapt the DNA extraction protocol. All so prepared sediment-enriched samples were filtered through Isopore 0.2 μm polycarbonate membrane filters (Millipore, Bedford, MA, United States) and filters were frozen immediately and stored at −80°C until nucleic acid extraction. An additional unused filter was placed directly into a 1.5 mL extraction vial as a blank filter control.

### Sampling for Test Series III—Comparison of DNA Extraction Protocols on Environmental Samples

Samples from Lake Neusiedl (L), the stabilization pond (P) and the wastewater treatment plant (W-ut and W-t) were taken monthly from April to December 2015. Two independent filtrations were done for each sample (surface water: 100 mL, un-treated waste water: 10 mL, treated waste water: 50 mL) using Isopore 0.2 μm polycarbonate membrane filters (Millipore, Bedford, MA, United States). On each sampling occasion, an additional unused filter was placed directly into a 1.5 mL extraction vial as a blank filter control. All filters were frozen immediately and stored at −80°C until DNA extraction. One replicate sample was extracted according to the standard DNA extraction protocol, the other one with a protocol adapted to the sample matrix (see sections “DNA Extraction” and “DNA Extraction Protocol Modification”). Both, the sample process control, DeTaCS, and salmon sperm DNA as adsorption site blocker, were added directly at DNA extraction into the reaction mixture. DeTaCS was added at a concentration of 5 × 10^6^ cells per extraction and salmon sperm DNA was added at an amount of 250 μg per reaction. Since TSS values were not available on a sample-to-sample basis the salmon sperm DNA amount for protocol modification was chosen based on the results of test series II. The here used amount would have been sufficient to compensate for 106 mg sediments L^–1^ sample.

### Chemo-Physical Water Analyses

Conductivity (LF 330, WTW, Germany), water temperature, pH (GHM, Seibold Vienna, Austria), oxygen (OXI 330i, WTW) and turbidity (calculated from Secchi depth) were measured *in situ*. For inorganic nutrients, TOC and TSS an extra water sample was collected in a clean 1 L plastic bottle and processed according to the methods published by Eiler et al. (2003). For the determination of TSS, a defined volume of sample water was filtered through pre-muffled glass-fiber filters (GF/C; Whatman, United Kingdom) and dried to constant weight at 120°C in a drying oven (Haereus, Germany). To obtain the inorganic and organic fraction of both suspended solids from water samples and lakebed sediments (uppermost 2–3 cm), the oven-dried samples were further combusted in a muffle furnace (480°C, 4 h; Nabertherm, Germany).

### Sample Process Control

The DeTaCS used as sample process control was an *Escherichia coli* strain (DHB6501) carrying a single copy of the target sequence for a ruminant-associated source tracking marker (BacR; Reischer et al., 2006; Mayer et al., 2018) in its genome. The strain was constructed by applying an *E. coli* plasmid-chromosome shuttle system using a λ-phage (Boyd et al., 2000). Details on strain construction as well as strains and plasmids used therefore are given as Supplementary Material (Supplementary Data 2). Production of the DeTaCS strain was done by batch fermentation in a benchtop bioreactor (RALF Plus-System, Switzerland). Details on fermentation conditions are given as supplementary Material (Supplementary Data 3). Aliquots were prepared of the fermentation batch to be used as process control to determine DNA extraction efficiency. For this purpose, culture broth was supplemented with glycerol to a final concentration of 20% and aliquots of 100 μL were shock frosted in liquid nitrogen and stored at −80°C for further use. Cell numbers were determined using an epifluorescence microscope (Nicon Eclipse 8000, Japan; see Supplementary Data 4). Samples spiked during filtration were supplemented with 5 × 10^7^ cells, samples spiked at DNA extraction directly were supplemented with 5 × 10^6^ cells per extraction.

### DNA Extraction

DNA extraction was performed using bead-beating and phenol/chloroform (Griffiths et al., 2000; Reischer et al., 2008; Mayer et al., 2018). In brief, cell lysis was achieved by addition of CTAB buffer and glass beads in a FastPrep 24 benchtop homogenizer for cell lysis (MP Biomedicals Inc., Irvine, CA, United States) at speed setting of 6 m s^–1^ for 30 s. Polycarbonate filters were completely dissolved at this step and the DNA was subsequently purified. Precipitation of the DNA was achieved by addition of isopropanol. The extracted DNA was eluted in 10 mmol L^–1^ TRIS buffer (pH 8.0) and stored at −80°C until further analysis. The detailed DNA extraction protocol is given as Supplementary Material (Supplementary Data 5).

### DNA Extraction Protocol Modification

Modification of the DNA extraction protocol to enhance DNA yield were direct addition of either salmon sperm DNA (0–65 mg g^–1^ sediment) or Na-pyrophosphate (0–0.5 g g^–1^ sediment) to the extraction buffer. The following extraction controls were prepared: blank extraction control (reagents only), extraction control spiked with DeTaCS (5 × 10^6^ cells per reaction), extraction control spiked with either salmon sperm DNA or sodium Na-pyrophosphate and an extraction control spiked with DeTaCS and either salmon sperm DNA or Na-pyrophosphate.

Salmon sperm DNA (Serva, Germany) added as adsorption site blocker was solubilized and purified using a standard phenol/chloroform extraction protocol. In brief, 500 mg salmon sperm DNA were solubilized in 50 mL sterile double-distilled water (4 h at room temperature on a rocking shaker; IKA, Germany) and NaCl concentration of the solution was adjusted to 0.1 M (Merck, Germany). DNA was extracted by addition of an equal volume of phenol (pH 7.5–8.0; Carl Roth, Germany). After centrifugation (5 min, RT, 13,000 rpm), the aqueous phase was transferred to a new reaction vial and extracted further by addition of an equal volume of phenol:chloroform (1:1). After centrifugation (5 min, RT, 13,000 rpm), the aqueous phase was again transferred to a new reaction vial containing an equal volume chloroform (Merck, Germany). After centrifugation (5 min, RT, 13,000 rpm), the aqueous phase was recovered and the DNA was sheared by passing it 10 times rapidly through a 20-gauge hypodermic needle (Braun, Germany). The recovered DNA was then precipitated by adding 2.5 volumes of ice-cold ethanol (96%; Merck, Germany). The solution was incubated at RT for 30 min. DNA was recovered by centrifugation (30 min, 4°C, 20.000 rpm). The obtained DNA pellet was finally dissolved in sterile double-distilled water and the final concentration was adjusted to 10 mg mL^–1^ salmon sperm DNA (Nanodrop 1000 spectrophotometer, Thermo Fisher Scientific). Before use as additive in the DNA extraction protocol, the DNA solution was tested for the absence of bacterial DNA contamination by 16S rRNA targeted qPCR (Klindworth et al., 2013). The obtained salmon sperm DNA solution was free of 16S rRNA gene targets.

Sample DNA concentration was determined with the QuantiFluor dsDNA Kit (Promega, United States) according to manufacturer’s instructions and fluorescence readings were taken on an Anthos Multimode Fluorometer Zenyth 3100 (UK-Biochrom Ltd.).

### Quantitative PCR

In addition to the sample process control DeTaCS (BacR assay; Reischer et al., 2006; Mayer et al., 2018), a general *Bacteroidetes* marker, AllBac (Layton et al., 2006), was run with the *ntb2* fragment as internal amplification control (IAC, non-competitive) in duplex to monitor for qPCR amplification inhibition (Anderson et al., 2011). qPCR methods targeting an enterococcal-associated genetic marker (ENT; Haugland et al., 2005) and the human-associated MST assay HF183/BacR287 (Green et al., 2014) were applied to test for the performance of the modified DNA extraction protocol on environmental samples.

All qPCR reactions were performed in duplicate in a 15 μL volume on a Rotor-Gene Q thermocycler (Qiagen Inc.). The reaction mixture for the AllBac and IAC duplex assay was composed of 7.5 μL Rotor-Gene Multiplex PCR mastermix (Qiagen Inc.), 2.5 μL sample DNA dilution (1:4 and 1:16), 600 nM AllBac296f forward primer, 600 nM AllBac412r reverse primer, 25 nM AllBac375Bhqr TaqMan MGB probe, 500 nM ntb2-f forward primer, 500 nM ntb2-r reverse primer, 200 nM ntb2-p probe and 400 ng μL^–1^ bovine serum albumin. IAC template (plasmid containing the *ntb2* gene fragment) was spiked at a concentration of 10^3^ copies per reaction. Cycling conditions were 3 min at 95°C for denaturation and 45 cycles of 30 s at 95°C followed by 45 s at 60°C. For the DeTaCS (BacR), HF183/BacR287 and the ENT assay the respective reaction mixture was composed of 7.5 μL Rotor-Gene Multiplex PCR mastermix (Qiagen Inc.), 2.5 μL sample DNA dilution (1:4) and 400 ng μL^–1^ bovine serum albumin, while the originally published primer and probe concentrations were maintained. Cycling parameters were 5 min at 95°C for denaturation and 45 cycles of 15 s at 95°C followed by 60 s at 60°C.

### Data Analysis

All data analysis was done with either Microsoft Excel for Mac 16.17 or Sigma Plot 10 (Systat Software Inc., Chicago, IL, United States). Quality assessment of qPCR data was done as previously described (Reischer et al., 2006, 2011; Mayer et al., 2018). In brief, reaction efficiency of all qPCR runs ranged from 95 to 105%. All negative controls and no-template controls were consistently negative (i.e., fluorescence never exceeded the threshold). All samples were measured in duplicate in at least two 4-fold DNA dilution steps with the AllBac assay and the results were compared. Samples with matching concentrations (e.g., the ratio [(concentration 1:16)⋅4]/[(concentration1:4)] was between 0.5 and 2) in the 1:4 and 1:16 dilutions were judged free of PCR inhibiting substances in the 1:4 dilution. This dilution was then used for all further measurements.

Samples with replicate standard deviations of a Ct-value > 1 in the four-fold DNA extract dilutions were considered to be not quantifiable and were not considered for further analysis. Moreover, an inhibition of the qPCR reaction was assumed to be present if the threshold cycle (Ct value) of the IAC assay in a sample was shifted toward higher Ct values by more than one cycle in comparison to the mean of the Ct of the negative controls. qPCR standard dilutions ranging from 10^1^ to 10^6^ targets per reaction were used in a linear regression model for calculation of the qPCR calibration curve. Results are reported as marker equivalents per DNA extract (ME per extraction, cf. Reischer et al., 2006).

Spearman’s rank-order correlation was used for calculation of the correlation coefficients among the parameters using SPSS Statistics Software version 25 (SPSS Inc., Chicago, IL, United States).

## Results

### Turbidity and Chemo-Physical Properties of Samples From the Study Area

The amount of suspended particles in a water sample is regularly given as either turbidity (NTU) or as TSS (mg L^–1^). In the present study, we provide values in both units to allow comparability with other studies. Turbidity and TSS values observed in the samples highlighted the high heterogeneity of the habitats within the study area in regard to the water matrix (Table 1). Turbidity values ranged between 3 and 341 NTU with the lowest values observed in treated wastewater and the highest ones in the soda lake (Oberer Stinker, OS > Lake Neusiedl, L > stabilization pond, P > reed belt, RB > treated wastewater, W-t). Values for TSS were highest in samples from the soda lake (OS) and lowest in samples from the reed belt (OS >> L ≥ P >> RB). The observed mean TSS values ranged from 4.6 mg L^–1^ (RB) to 3,015.5 mg L^–1^ (OS).

**TABLE 1:** Chemo-physical parameters of environmental water samples from the six different sampling sites within the study area.

		**Turbidity**	**TSS**	**TOC**	**Conductivity**	**pH**
		**(NTU)**	**(mg L^–1^)**	**(mg L^–1^)**	**(μS cm^–1^)**	
OS	**Mean**	**340.6**	**3,015.5**	**39.1**	**4,202.0**	**9.3**
	Range	70.0–650.0	1,739.7–3,307.8	31.2–47.0	2,980.0–6,000.0	9.1–9.5
L	**Mean**	**162.0**	**24.2**	**12.0**	**1,609.6**	**8.8**
	Range	31.0–455.0	22.3–26.4	11.2–12.5	1,456.0–1,842.0	8.7–8.9
RB	**Mean**	**3.2**	**4.6**	**22.3**	**1,878.6**	**8.6**
	Range	1.6–5.3	3.4–5.6	18.0–26.5	1,736.0–2,080.0	8.4–9.0
P	**Mean**	**11.2**	**21.2**	**5.8**	**994.5**	**7.9**
	Range	2.2–26.0	10.2–43.6	3.9–7.1	780.0–1,111.0	7.7–8.2
W-ut	**Mean**	n.a.	**286.5**	n.a.	n.a.	n.a.
	Range	n.a.	175.4–472.4	n.a.	n.a.	n.a.
W-t	**Mean**	**2.8**	**5.1**	**6.0**	**1,009.2**	**7.6**
	Range	1.6–4.2	5.0–5.2	4.4–10.6	811.0–1,138.0	7.3–7.8

*OS, soda lake Oberer Stinker, L, Lake Neusiedl; RB, reed belt of Lake Neusiedl; P, wastewater stabilization pond; W-ut, untreated wastewater; W-t, treated wastewater; NTU, Nephelometric Turbidity Units; TOC, Total Organic Carbon; TSS, total suspended solids.*

*n = 5–11.*

In water samples, the organic fraction of TSS ranged from 3.9 to 97.1% (P >> RB ≥ L >> OS) while the inorganic fraction ranged from 2.9 to 96.1% (OS >> L ≥ RB >> P; Table 2). In sediment samples, the mean organic fraction ranged from 0.6% in Lake Neusiedl to 16.2% in the reed belt (RB > P >> OS > L) and the mean inorganic fraction ranged from 83.8% in the reed belt to 99.4% in Lake Neusiedl (L > OS >> P > RB).

**TABLE 2:** Percentage of the organic and inorganic fraction of suspended solids from water samples and lakebed sediment for environmental samples from Soda Lake (OS), Lake Neusiedl (L), the Reed Belt (RB), and the stabilization pond (P).

		**OS**		**L**		**RB**		**P**	
		**Water**	**Sed.**	**Water**	**Sed.**	**Water**	**Sed.**	**Water**	**Sed.**
Organic component (%)	**Mean**	**3.9**	**1.5**	**43.2**	**0.6**	**45.8**	**16.2**	**97.1**	**13.7**
	Median	4.1	1.5	41.6	0.5	47.8	16.5	100	9.9
	Min	2.7	1.1	30.8	0.4	28.8	13.8	91.4	7.2
	Max	5.0	1.8	63.9	0.8	62.2	17.8	100	35.2
Inorganic component (%)	**Mean**	**96.1**	**98.5**	**56.8**	**99.4**	**54.2**	**83.8**	**2.9**	**86.3**
	Median	95.9	98.5	58.4	99.5	52.2	83.6	0.0	90.1
	Min	95.0	98.2	36.1	99.2	37.8	82.2	0.0	64.8
	Max	97.3	98.9	69.2	99.6	71.2	86.2	8.6	92.8

*n = 6. Min, minimum; max, maximum; Sed., sediment.*

Total organic carbon values ranged from 6 to 39 mg L^–1^ with the lowest values observed in the stabilization pond and the highest ones in the Soda Lake (P < W-t < L < RB < OS). The open lake was therefore mainly characterized by high turbidity and lower TOC, while the reed belt was characterized by low turbidity and high TOC. The soda lake represented an extreme habitat in every respect (high turbidity and TOC levels).

Electrical conductivity in the different sample types from the study area ranged between 1,000 and 4,200 μS cm^–1^ (P < L < RB < OS) and the pH value was ≥8 (Table 1). The lowest pH values were found in the stabilization pond and in treated wastewater (pH 8), the highest pH values were observed in the soda lake (pH ≥ 9, Table 1).

### Collecting Field Evidence That Chemo-Physical Parameters Influence DNA Extractability

**FIGURE 1:**
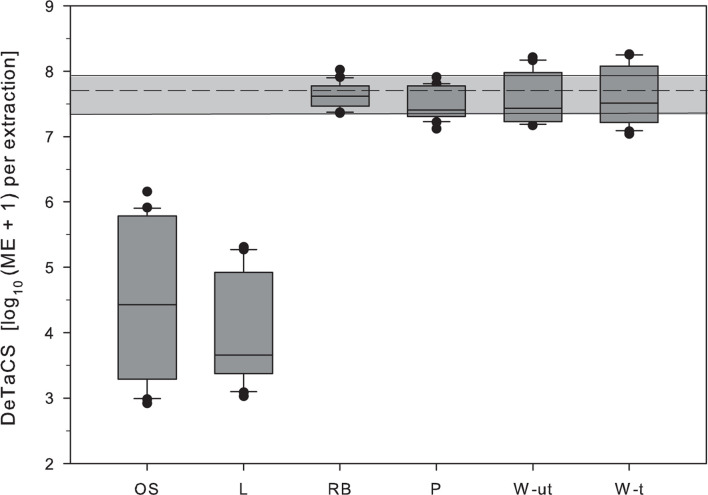
Retrieval of DeTaCS marker concentrations from environmental samples of the study area to test the hypothesis that the sample matrix can affect DNA extraction efficiency. Environmental water samples were spiked with the reference cell standard during filtration directly into the sample. The given sample order reflects the gradient from high to low inorganic TSS fraction (OS > L >> RB > P > W-ut > W-t). OS, shallow soda lake Oberer Stinker; L, Lake Neusiedl; RB, reed belt; P, stabilization pond; W-ut, untreated wastewater; W-t, treated wastewater. Boxes, 25th and 75th percentile; lines within the boxes, median; whiskers, 10th and 90th percentile, respectively. The gray area spans the 10th–90th percentile of the DeTaCS marker concentration in the extraction controls. The dashed line marks the median. *n* = 22.

As a measure of DNA extraction efficiency from environmental samples of the study area the recovery of the added process control (DeTaCS) was used. Losses observed during sample processing were most pronounced in samples from Lake Neusiedl (L) and the shallow soda lake (OS). In these two sample types, the marker concentrations retrieved were lower by a factor of 4 and 2 log_10_ units when compared to spiked controls, respectively. Both sample types were characterized by high turbidity values and a high percentage of inorganic compounds (Tables 1, 2). In the other sample types (RB, P, W-t), the DeTaCS marker was detected in a concentration range similar to the extraction controls (Figure 1). A correlation analysis revealed a significant negative relationship of the DNA extraction efficiency with turbidity and TOC levels (turbidity: ρ = −0.719, *p* < 0.001, *n* = 24; TOC: ρ = −0.407, *p* = 0.048, *n* = 24, Supplementary Table 3). PCR inhibition, as would have been indicated by a shift of the IAC toward higher Ct values, could not be observed in any of the sample types. Observed Ct values in controls ranged from 30 to 31, those of samples ranged from 29 to 31.

### Laboratory Testing of the Hypothesis

To further investigate the possible effects of the water sample matrix on DNA yield, samples were prepared by suspending lakebed sediment in lake water (equivalent to a TSS gradient covering a range of 9–220 mg L^–1^). These sediment-enriched samples were then subjected to DNA extraction and the DNA yield and the AllBac marker concentration were subsequently determined to investigate possible effects of the suspended particles. The AllBac qPCR assay was chosen because it targets a broad range of bacteria of the *Bacteroidetes* phylum including target organisms of many MST assays.

**FIGURE 2:**
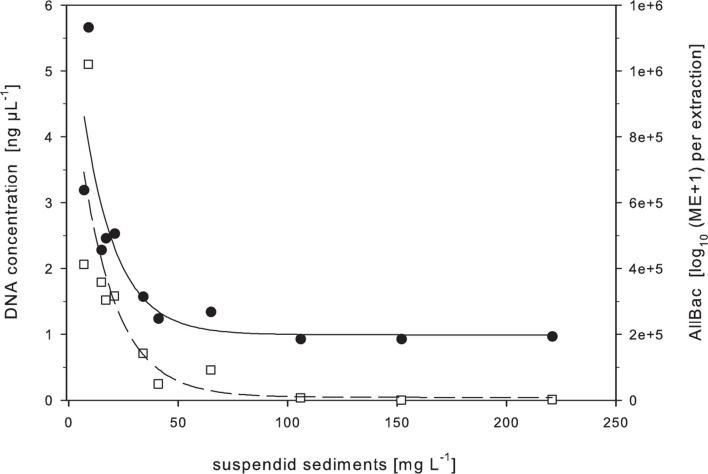
DNA yield (black circles) and AllBac marker concentrations (white squares) in dependence of in lake water suspended lakebed sediments. ME, marker equivalents. *n* = 1 per sediment concentration.

The results (Figure 2) showed that sediment concentrations of 15 mg L^–1^ already reduced the DNA retrieval to approximately 43% (2 ng μL^–1^ DNA). Sediment concentrations of 40 mg L^–1^ and above resulted in a retrieval of approximately 20% (1.0–1.3 ng μL^–1^ DNA). AllBac marker concentrations obtained from this sample set reflected the same TSS dependent trend as observed for the DNA concentration. A spearman rank order correlation confirmed the significance of the negative correlation between the sediment content and the retrieved DNA concentration (ρ = −0.916, *p* < 0.001, *n* = 11, Supplementary Table 4) as well as the retrieved AllBac marker concentration (ρ = −0.964, *p* < 0.001; *n* = 11, Supplementary Table 4).

### Optimization of the DNA Extraction Protocol by Addition of Adsorption Site Blockers

The results obtained from the previous experiments indicate that it is mainly those sample types with a high fraction of inorganic matter in which DNA extraction efficiency is strongly reduced. Since these particles are often characterized by high adsorptive capacities toward nucleic acids (see section “Discussion”), a DNA extraction protocol optimization was endeavored by testing two frequently used adsorption site blockers, salmon sperm DNA and Na-pyrophosphate.

**FIGURE 3:**
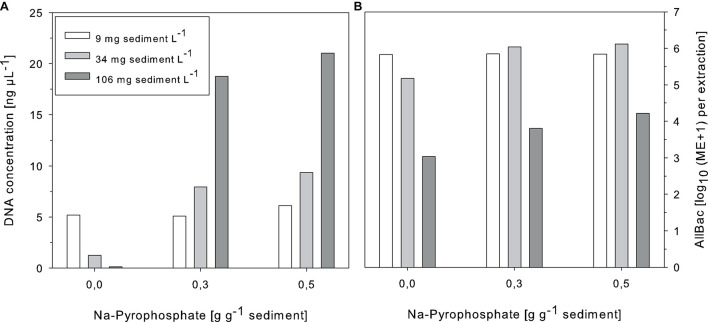
Effects of Na-pyrophosphate supplementation during DNA extraction on the DNA yield **(A)** and AllBac marker concentrations **(B)** of sediment-enriched samples. ME, Marker equivalents, *n* = 1.

In a first attempt Na-pyrophosphate was supplemented during DNA extraction as it would have the advantage not to bring in DNA into the sample. The amounts needed to compensate for adsorptive effects were high (>0.3 g g^–1^ sediment) but the DNA retrieval could be significantly increased (Figure 3). In samples with low sediment concentration Na-pyrophosphate addition did not affect DNA yield while for samples with higher sediment content (34 and 106 mg L^–1^) no saturation plateau in the DNA increase could be observed. AllBac marker concentrations also increased after supplementing the reaction mixture with Na-pyrophosphate. However, in samples with the highest amounts of suspended sediments AllBac marker concentrations were not raised to the same extent as the DNA yield (Figure 3). In these samples, a strong inhibition of the qPCR reaction was observed as measured by application of the IAC. A shift in the Ct value of 2–4 cycles was observed in these samples for which reason Na-pyrophosphate was omitted from the spiking- and all further experiments.

**FIGURE 4:**
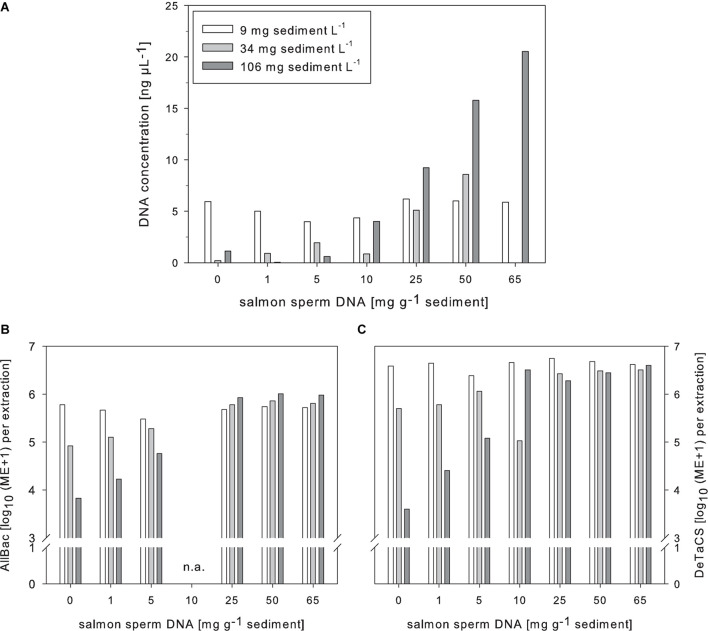
Effects of salmon sperm DNA supplementation during DNA extraction on the DNA yield **(A)**, the AllBac marker concentration **(B)**, and the DeTaCS marker concentrations **(C)** of sediment-enriched samples. ME, Marker equivalents, *n* = 1.

The addition of salmon sperm DNA during the extraction process also strongly increased the DNA retrieval and the effect was greater the higher the sediment content was (Figure 4). Like for the DNA yield, the AllBac marker concentration also increased after salmon sperm DNA addition. The increase was most pronounced in samples with the highest sediment content tested (106 mg L^–1^) while the marker concentrations in samples with the lowest sediment concentration (9 mg L^–1^) remained merely unchanged. Further, a saturation plateau for the AllBac marker concentration after salmon sperm addition was observed in samples with higher sediment concentrations (34 and 106 mg L^–1^). The amount of salmon sperm DNA needed to reach this point, however, was different (Figure 4).

Supplementing the samples with salmon sperm DNA and additionally spiking with the DeTaCS revealed a picture comparable to that obtained for the AllBac marker. The DeTaCS marker concentrations could also be significantly increased due to supplementation with salmon sperm DNA and, as for the AllBac marker, a saturation plateau was reached after which further salmon sperm DNA addition did not affect the retrieved DeTaCS marker concentrations further. Significant correlations were obtained for the amount of added salmon sperm DNA with the DNA concentration retrieved (ρ = 0.712, *p* < 0.001, *n* = 21) as well as the DeTaCS marker concentration recovery (ρ = 0.515, *p* = 0.017 *n* = 21; Supplementary Table 5).

### Impact of the Modified DNA Extraction Protocol on Molecular Fecal Pollution and MST Marker Results

**FIGURE 5:**
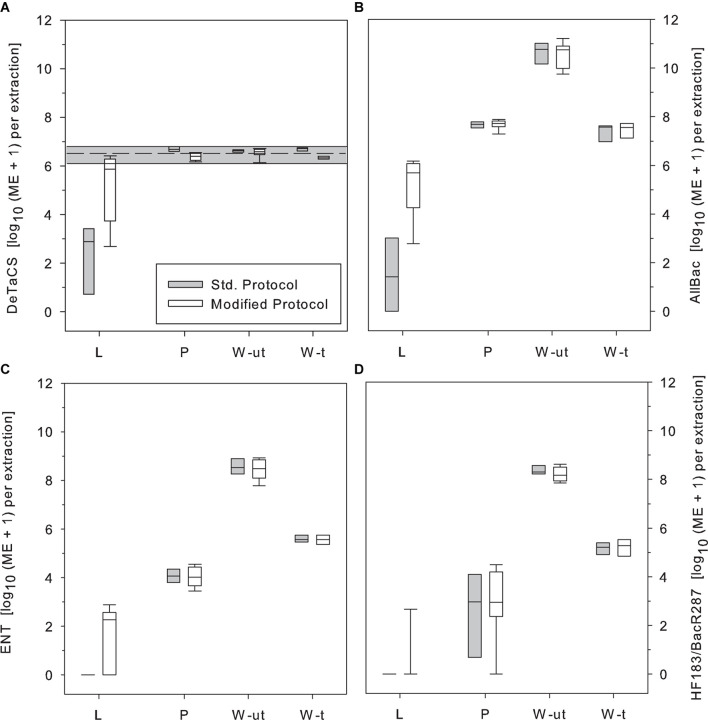
Impact of the applied DNA extraction protocol (standard extraction protocol, gray; addition of salmon sperm DNA, white) on the retrieval of the sample process control (DeTaCS) **(A)**, the AllBac marker concentration **(B)**, the qPCR-based ENT marker **(C)**, and the human-asscoiated MST marker HF183/BacR287 results **(D)**. Std. Protocol, standard DNA extraction protocol; Modified Protocol, Supplementation of the reaction mixture with salmon sperm DNA during DNA extraction; L, Lake Neusiedl; P, stabilization pond; W-ut, untreated wastewater; W-t, treated wastewater. Boxes, 25th and 75th percentile; lines within the boxes, median; whiskers, 10th and 90th percentile, respectively. *n* = 8–9.

Finally, the standard and the modified DNA extraction protocols were tested in comparison on environmental samples from the study area. The strongest effect of protocol modification became obvious in samples from Lake Neusiedl (L). In this sample type, the addition of salmon sperm DNA raised DNA extraction efficiency to close to control levels in the majority of the samples while the extraction efficiency of the other sample types tested (P, W-t, W-ut) remained merely unchanged. In samples from Lake Neusiedl, however, the DeTaCS marker concentrations retrieved with the modified protocol were, on average, 3 log_10_ units higher as with the standard procedure (Figure 5A).

When applied to molecular genetic fecal markers and MST markers, the benefit of using the modified protocol as opposed to the standard extraction protocol became especially clear. Again, the greatest differences were observed for samples from Lake Neusiedl. In this sample type, the median AllBac marker concentration increased by 4 log_10_ steps due to the addition of salmon sperm DNA as adsorption site blocker during DNA extraction (median standard protocol: log_10_ 1.4 ME 100 mL^–1^, median modified protocol: log_10_ 6.0 ME 100 mL^–1^, Figure 5B).

Particularly striking were the effects observed on the applied qPCR-based ENT marker and the human-associated MST marker HF183/BacR287. Since these markers are often present in the environment at very low concentrations, they were not detectable after extraction with the standard protocol. Only after application of the modified extraction protocol these genetic markers could also be detected in many samples (Figures 5C,D). The qPCR-based ENT marker, for example, which could not be detected at all after DNA extraction with the standard protocol, was found at median concentrations of log_10_ 1.7 ME 100 mL^–1^ (Figure 5C) after addition of salmon sperm DNA during extraction. The HF183/BacR287 marker was also not detected in samples extracted with the standard protocol but was found sporadically after extraction with the modified protocol (Figure 5D).

## Discussion

Many studies investigating microbiological water quality have addressed the issue of inhibitory effects of the sample matrix in the qPCR reaction which is routinely determined by the use of an internal amplification control (e.g., Gregory et al., 2006; Behets et al., 2007; Sen et al., 2007; Shanks et al., 2008; Haugland et al., 2010; Anderson et al., 2011; D’Agostino et al., 2011). Possible DNA losses during sample processing (filtration, DNA extraction) which might occur independently of PCR inhibition, remain undetected and unconsidered. This also became obvious from the results of the present study, where in neither of the sample types an inhibitory effect on the qPCR reactions was observed despite massive losses of DNA during the extraction process, highlighting the importance of sample process controls by which these effects could be uncovered in the present study.

However, from a large number of studies, mainly in the research field of soil microbiology, it has been shown that soil and sediment particles can interfere with DNA extraction efficiency (e.g., Lombard et al., 2011; Yu et al., 2013; Lever et al., 2015; Gardner and Gunsch, 2017; Xue and Feng, 2018). In studies on water quality analysis this aspect has only recently gained attention (Haugland et al., 2012; Shanks et al., 2016; Li et al., 2019; Rocha and Manaia, 2020) although it seems reasonable to assume that the mechanisms responsible for DNA losses in soil samples (organic and inorganic particles that have the capacity to adsorb nucleic acids during the process of DNA extraction) might also become active in water samples characterized by high amounts of suspended solids. In a first step this hypothesis was tested in the present study on environmental samples from an aquatic habitat covering a strong gradient of chemo-physical parameters (turbidity, TSS). The results clearly supported this hypothesis by showing a non-negligible effect of both, turbidity and TSS, on DNA extraction efficiency, with the greatest losses observed in samples with high inorganic content (L, OS; Figure 1) and less or no effect on other sample types (lower levels of inorganic particulate matter or higher levels of organic materials) as shown by application of the sample process control. This finding is in line with other studies that have shown that losses of DNA during the extraction process are directly related to the soil or sediment’s composition (Frostegard et al., 1999; Miller et al., 1999; Lombard et al., 2011; Paulin et al., 2013). According to these studies, it is mainly those samples with a high clay content (Cai et al., 2006; Yu et al., 2013; Lever et al., 2015; Engel et al., 2019) to which DNA adsorbs and/or binds particularly well after its release from the cell. The therefore underlying mechanism is mainly explained as adsorption of DNA to inorganic particles by bridging effects exerted by divalent cations between phosphate groups of the DNA and the silicate anions of the minerals (Lorenz and Wackernagel, 1994; Saeki et al., 2010). Since the lakebed sediment of Lake Neusiedl consists mainly of fine-grained clays and carbonates interrupted by thin sand layers and small gravel (Löffler, 1979; Hedrich, 1983) an effect seems reasonable. Sand and silt contents of the lake sediment predominantly consist of dolomite, calcite, quartz, mica, and oligoklases while clay minerals are mainly composed of illite and, to a lesser extent, montmorillonite (Löffler, 1979). Further studies on laboratory-prepared samples with varying TSS content strengthened the hypothesis that inorganic turbidity strongly influences DNA extraction efficiency by showing a strict dependence between the amount of suspended lake bottom sediment and the recovered DNA concentration (presented as DNA concentration and AllBac marker equivalents, Figure 2).

While the assessment of DNA extraction efficiency is crucial for unraveling detrimental effects of the sample matrix, for some applications, especially when the results are used to inform critical decision-making, there may be a further need to adapt protocols to improve extraction efficiency. For this purpose, today a plethora of different protocols to extract DNA from difficult sample matrices is available (e.g., Paulin et al., 2013; Lever et al., 2015; Hinlo et al., 2017; Engel et al., 2019). Most protocol modifications were made by using additives such as phosphate and inorganic phosphate types, nucleic acid building blocks, DNA, RNA, or skim milk powder (e.g., Frostegard et al., 1999; Hoshino and Matsumoto, 2005; Cai et al., 2006; Paulin et al., 2013; Lever et al., 2015) to coat charged surfaces before cell lysis. The choice of additives used, however, may depend on their availability and/or cost as well as on the research question posed. Using milk powder, for example, could raise the question whether traces of bacterial DNA could be introduced influencing the PCR reaction or affecting results in microbiome studies (Schrader et al., 2012; Paulin et al., 2013). In contrast, the use of salmon sperm DNA is only applicable if not simultaneously used as sample process control (e.g., Sketa Assay, U.S. EPA, 2010).

In the present study, salmon sperm DNA and Na-pyrophosphate were tested as additives. Both significantly increased DNA extraction efficiency. In the case of Na-pyrophosphate the high doses used were not able to compensate for the adsorptive capacities of the suspended matter since no saturation plateau was reached for the DNA concentration retrieved. However, in some samples negative effects of Na-pyrophosphate addition were observed in form of PCR inhibition (shift in the Ct value > 1 compared to controls), which is why the Na-pyrophosphate addition was no longer followed up in this study. This observation is also in line with that of Lever et al. (2015) who observed that DNA pellets retrieved after Na-pyrophosphate addition were not only larger but also darker in color, indicating an increased transfer of non-nucleic acid containing organic matter (e.g., humic substances) to the eluate which could further lead to PCR inhibition, as it was observed also in the present study.

The results obtained after salmon sperm DNA addition were somewhat different. As for Na-pyrophosphate addition no saturation plateau of the DNA concentration was retrieved. For the DeTaCS and AllBac marker concentrations, however, indeed a saturation plateau was reached, albeit the amounts of salmon sperm DNA needed to compensate for the adsorptive effects were very high compared to other studies. In general, the quantities of various DNAs described in the literature to be needed for adsorption site saturation vary greatly (e.g., Paulin et al., 2013; Lever et al., 2015; Gardner and Gunsch, 2017; Engel et al., 2019) as do the adsorption capacities described for different sediment types themselves. There seems no consensus conclusion regarding what type of clay exhibits the strongest adsorption capacity for nucleic acids and experimental setup of studies published vary greatly, making a direct comparison difficult. However, both illite and montmorillonite, which are present in the water bodies of Lake Neusiedl area (Löffler, 1979), are described as highly adsorptive for DNA (Ben-Hur et al., 1992; Poly et al., 2000; Cai et al., 2006; Saeki et al., 2010; Yu et al., 2013).

Finally, the established hypothesis was tested on environmental samples from the habitat. To evaluate possible and, based on the previous results, expected effects on quantitative results of selected molecular fecal and MST markers, DNA extraction was performed using the standard and a modified DNA extraction protocol, respectively. For the above mentioned reasons, in the comparative tests solely salmon sperm DNA was used as supplement. However, possible adverse effects on the measurements of DNA concentration should be considered as there might be competitive reactions of salmon sperm DNA and sample DNA. Both will compete for free charges, both will bind to them and it must not be assumed that all adsorption sites are fully saturated before cells are broken up and sample DNA is released from the cells. For this reason, the addition of salmon sperm DNA will not necessarily raise sample DNA retrieval to control levels since some sample DNA will also bind to free charges on particle surfaces. This was also observed in the present study. The DeTaCS retrieval was raised by three log_10_ units but still remained below the control level (Figure 3A). Furthermore, excess amounts of salmon sperm DNA could be co-precipitated alongside with sample DNA affecting the reliability of (sample) DNA concentrations determined (Paulin et al., 2013).

Despite all these considerations the comparative application of the two extraction protocols impressively showed the influence on the obtained quantitative results (Figures 3A–D). As in all previous tests the strongest influence of the extraction method was observed in samples from Lake Neusiedl. Both, the qPCR-based ENT marker and the human-associated MST marker HF183/BacR287, could not be detected in any sample subjected to standard DNA extraction. Only after application of the modified extraction protocol these markers could also be detected (Figures 3C,D), a result which underlines the importance of testing for matrix effects and possibly applying an adapted extraction protocol. These results, therefore, not only support the hypothesis that inorganic turbidity, when present, can affect the efficiency of DNA extraction, but also clearly demonstrate the influence on the ability of molecular methods to yield reliable quantitative data. Routine use of these markers to determine microbiological water quality would not have revealed, at least intermittently, fecal contamination in the study area if the standard extraction protocol had been used. In cases where such studies are used for risk assessment and targeting of management measures (e.g., guidance on closure of bathing sites), a proper assessment of the current situation would not be accurate.

## Conclusion

The results of the present study clearly indicate that difficulties in the preparation of environmental samples due to matrix effects, as known from soil samples, should also be considered in the processing of water samples. Further, it seems that it is more the inorganic fraction of suspended solids which influences DNA extraction efficiency since in all experiments DNA retrieval from samples with high inorganic turbidity was affected most severely. Since these materials are present in many water bodies, this factor might become effective in a variety of sample types and even more if samples are taken under certain environmental conditions. By this, samples taken e.g., during flood events and/or strong wind events might be particularly vulnerable to the occurrence of such effects (whirling up of sediments). However, whether effects on DNA extraction are to be expected will not only depend on the amount of suspended material but also on its composition which in turn might affect the amount of additives required to improve the extraction efficiency. For this reason no universal DNA extraction protocol or general rule of how to improve extraction efficiency can be given.

Apart from all considerations presented here, however, the main conclusion to be drawn is the immense importance of the use of controls, irrespective if further measures are taken in attempt to optimize an extraction protocol. The use of process controls (filtration and extraction controls) on a sample-to-sample basis, will allow direct conclusions on the extraction efficiency. As a more indirect measure, the monitoring of turbidity and TSS could be anticipated, whereby it should be borne in mind that different sediment types can differ strongly with their respect of influencing DNA extraction efficiency.

## Data Availability Statement

The original contributions presented in the study are included in the article/Supplementary Material, further inquiries can be directed to the corresponding author.

## Author Contributions

RL: investigation, laboratory work, writing—original draft, and writing—review and editing. SZ and ReM: investigation, laboratory work, and writing—original draft. KK: DeTaCS construction and writing—original draft. DS: investigation and writing—original draft. AK: conceptualization, funding acquisition, and writing—original draft. GR: conceptualization and writing—original draft. RoM: conceptualization. RS: conceptualization and funding acquisition. AF: conceptualization, writing—original draft, writing—review and editing, funding acquisition, and supervision. All authors contributed to the article and approved the submitted version.

## Conflict of Interest

The authors declare that the research was conducted in the absence of any commercial or financial relationships that could be construed as a potential conflict of interest.

## Publisher’s Note

All claims expressed in this article are solely those of the authors and do not necessarily represent those of their affiliated organizations, or those of the publisher, the editors and the reviewers. Any product that may be evaluated in this article, or claim that may be made by its manufacturer, is not guaranteed or endorsed by the publisher.
