# Supplementary material for: Identifying Inorganic Turbidity in Water Samples as Potential Loss Factor During Nucleic Acid Extraction: Implications for Molecular Fecal Pollution Diagnostics and Source Tracking
Source: Front Microbiol. 2021 Oct 20;12:660566. doi: 10.3389/fmicb.2021.660566 (PMC8565874; doi:10.3389/fmicb.2021.660566)
Supplement: Supplementary file 1 [file Data_Sheet_1.docx]

**Supplementary Material**

**Supplementary Data 1: preparation of dilution series of lakebed sediment in lake water**

For the preparation of the sediment-enriched samples, different amounts of lake bottom sediment were suspended in water from Lake Neusiedl. The table below gives the mixing ratios and the corresponding TSS values. For the evaluation of the dependence of the DNA concentration on the TSS content all prepared samples were used. Finally, for the follow-up experiments (influence of the applied extraction protocol on the DNA yield), only the samples 1, 7 and 10, corresponding with 9, 34 and 106 mg sediment per L were used.

**Supplementary Data Table S1:** Overview of the mixing ratios of sediment suspension and lake water used to prepare the sediment-enriched samples for Test Series II. Samples used for the follow-up experiments (influence of the extraction protocol on the DNA yield) are highlighted in bold.

|  | **Lake water [mL]** | **Sediment suspension [mL]** | **TSS [mg / L]** |
| --- | --- | --- | --- |
| **Sample 1** | **100** | **0** | **9** |
| Sample 2 | 98 | 2 | 9 |
| Sample 3 | 96 | 4 | 7 |
| Sample 4 | 94 | 6 | 17 |
| Sample 5 | 92 | 8 | 21 |
| Sample 6 | 90 | 10 | 15 |
| **Sample 7** | **85** | **15** | **34** |
| Sample 8 | 80 | 20 | 41 |
| Sample 9 | 70 | 30 | 65 |
| **Sample 10** | **50** | **50** | **106** |
| Sample 11 | 30 | 70 | 152 |
| Sample 12 | 0 | 100 | 221 |

**Supplementary Data 2: Strain construction of sample process control**

The defined target cell standard (DeTaCS) used as sample process control in the present study is an *Escherichia coli* strain (DHB6500) carrying the target sequence for a qPCR assay as a single copy in its genome. As target for the qPCR assay the readily available BacR assay (Reischer *et al.,* 2006) was used. For strain construction, an *E. coli* plasmid-chromosome shuttle system using a λ phage was used (Boyd *et al.,* 2000). This system was established for generating genomic integration of plasmid-encoded genes as single-copy integration in *E. coli* by using a bacteriophage λ derivative named lambda InCh2. The target sequence for the BacR assay was amplified from ruminant fecal samples using the primers BacR_f (GCGTATCCAACCTTCCCG) and BacR_r (CATCCCCATCCGTTACCG) targeting the 16S rDNA. The PCR product was purified using PROMEGA Wizard®-SV Gel and PCR Clean-Up System (Promega, Madison, USA) and ligated in a pGEM®-T easy vector (Promega, Madison, USA) which was then transformed in chemically competent *E. coli* Top10 cells. The cloned sequence was sequenced by MWG-Biotech (Ebersberg, Germany) using universal primers SP6 and T7. For genomic sequence integration in *E. coli* the plasmid carrying the BacR sequence was transformed in electro-competent *E. coli* DHB6500 cells carrying the bacteriophage λ derivative lambda InCh2. Genomic integration of the BacR sequence in *E. coli* DHB6501 required three successive *in vivo* steps involving both homology-dependent as well as site-specific recombination. The steps required for this process were (i) recombination of the desired plasmid-encoded sequence onto lambda InCh2 in *E. coli*; (ii) integration of the recombinant lambda InCh2, carrying the newly incorporated sequence, into the chromosome at the lambda attachment site; and (iii) deletion of most of the lambda genes, including one attachment site, from the chromosome of the lysogen (Boyd *et al.,* 2000). Strains and plasmids for the construction of the DeTaCS strain are summarized in Supplemetary Data Table S1. The required *E. coli* recipient strains, *E. coli* DHB6500 and *E. coli* DHB6501, were kindly gifted to our working group by D. Boyd.

**Supplementary Data Table S2:** Strains and plasmids used for DeTaCS construction.

| **Strain or plasmid** | **Relevant genetic marker(s) or features** | **Source or reference** |
| --- | --- | --- |
| *Strains* |  |  |
| DHB6500 | DHB6501 λInCh2 (Kan^r^) | kindly gifted from D. Boyd |
| DHB6501 | F^-^ λ^-^ λ^s^ *Δlac(MS265)* *mel* NalA^r^ *supF58*(=*suIII^+^*) | kindly gifted from D. Boyd |
| *Plasmids* |  |  |
| DeTaCS_BacR | BacR target sequence in pGEM®-T easy | This study |

**Supplementary Data 3: Production of the DeTaCS strain**

Production of the DeTaCS strain was done by batch fermentation of 2.0 L in a benchtop bioreactor (RALF Plus-System, Bioengineering AG, Switzerland). Fermentation was performed in LB medium (peptone 10 g L^-1^, NaCl 10 g L^-1^, yeast extract 5 g L^-1^) containing ampicillin (Carl Roth, Germany) at a concentration of 25 µg mL^-1^ at 21°C. The low growth temperature was chosen to prolong generation time since *E. coli* is capable of initiating genome replication for the next cell division round before the current cell division ended (Cooper *et al.*, 1968). For the use as process control, however, the existence of only one copy per cell, if possible, is preferred. The pH was set to 7 and controlled by addition of 3 M KOH and 3 M HCl, respectively. Stirring speed was set to 500 rpm and air flow was set to 100 NL h^-1^. Fermentation was documented by recording a growth curve by taking hourly samples and determining the OD_600_ spectrophotometrically (JASCO Corporation, Hachioji City, Japan) of the culture. Cells were harvested at the mid-exponential growth phase. Culture broth was supplemented with glycerol to a final concentration of 20% and aliquots of 100 µL were shock frosted in liquid nitrogen and stored at -80°C for further use.

**Supplementary Data 4: Determination of DeTaCS cell density in spiking aliquots**

For determining the cell concentration of the aliquots, cells of six aliquots were diluted 1000-fold in sterile water (Merck, Germany) and fixed in 4% formaldehyde (Merck, Germany) for one hour at 4°C. Cells from one mL of these dilutions were collected by filtration over a 0.2 µm Anodisc filter (Whatman, Maidstone, UK) and stained with 30 µL of a 400-fold dilution of SYBR gold (Thermo Fisher Scientific, Waltham, USA) at room temperature for 17 min in darkness. Cells numbers were determined using an epifluorescence microscope (Nikon Eclipse 8000, Japan). Images were captured with a Nikon DS-QiMc camera and edited using NIS Elements BR 2.3 software (Nikon, Tokyo Japan). From each sample, cells of ten different areas of each 15625 µm^2^ were counted. The cell density amounted to 5 x 10^5^ cells µL^-1^.

**Supplementary Data 5: Detailed DNA extraction protocol**

Glass beads (VWR International) used for DNA extraction were placed in a muffle furnace at 400°C for 2 hours. Screw-vials of 2 mL volume (VWR International) were filled each with one glass bead of 5 mm diameter, 0.27g glass beads of 1 mm diameter and 0.37g of glass beads of 0.1 mm diameter. All so prepared reaction tubes were autoclaved (121°C, 20 min) before use. For DNA extraction, each reaction vial was filled with 400 µL phenol (Carl Roth, Germany), 400 µL CTAB buffer (AppliChem, Germany) and 400 µL of chloroform/isoamyl alcohol 24:1 (AppliChem, Germany) before sample filters were added. In case samples were spiked with DeTaCS and / or salmon sperm DNA, both were added to the reaction vial after placing the sample filter in the vial. Cell lysis was subsequently achieved by placing the reaction vials in a FastPrep 24 benchtop homogenizer for cell lysis (MP Biomedicals Inc., Irvine, CA) at speed setting of 6 m s^-1^ for 30 s. Thereafter, samples were immediately placed on ice (1 min). Samples were then centrifuged at 13.000 g at room temperature for 5 min. The supernatant (approximately 500 µL) was transferred to a fresh 1.5 mL LoBind reaction vial (Eppendorf, Germany) containing 500 µL chloroform/isoamyl alcohol 24:1. Samples were vortexed vigorously and centrifuged again at 13.000 g at room temperature for 5 min. The supernatant (approximately 450 µL) was transferred to a fresh 1.5 mL LoBind reaction vial containing 270 µL isopropanol (Merck, Germany). Mixing was done by inverting the reaction vials. Samples were centrifuged at 13.000 g at room temperature for 15 min. The supernatant was discarded and 1 mL of ice cold ethanol (70%) was added to the reaction vial. Samples were centrifuged at 13.000 g at 4°C for 5 min. The supernatant was removed with a pipette and reaction vials were left open over night at room temperature protected with a cover. DNA pellets were resuspended by addition of 100 µL 10 mM TIS (pH8). All DNA extracts were stored at -80°C until further analysis. All pipetting steps were performed using LoBind tips (Sorenson; USA).

**Supplementary Data Table S3.** Spearman rank order correlation results for chemo-physical parameters and DNA concentration, AllBac [log_10_ (ME+1) per extraction] and DeTaCS [log_10_ (ME+1) per extraction]. Data from all sampling sites within the study area (OS, L, RB, P, W-ut, W-t) were pooled for this analysis. *: The correlation is significant at the 0.05 level, **: The correlation is significant at the 0.01 level, n = 24. *Abbreviations:* TSS, Total Suspended Solids; TOC, Total Organic Carbon; Cond., Conductivity; DNA, DNA concentration; DeTaCS, Defined Target Cell Standard (sample process control).

|  |  | **TSS** | **TOC** | **Cond.** | **pH** | **DNA** | **AllBac** | **DeTaCS** |
| --- | --- | --- | --- | --- | --- | --- | --- | --- |
| **Turbidity** | ρ | 0.804** | 0.516** | 0.442* | 0.737** | -0.265 | -0.430* | -0.719** |
| **[NTU]** | significance | <0.001 | 0.010 | 0.003 | <0.001 | 0.245 | 0.036 | <0.001 |
| **TSS** | ρ | 1.000 | 0.577* | 0.282 | 0.689** | 0.329 | 0.311 | -0.336 |
| **[mg L^-1^]** | significance |  | 0.012 | 0.257 | 0.002 | 0.182 | 0.170 | 0.136 |
| **TOC** | ρ |  | 1.000 | 0.767** | 0.887** | 0.019 | -0.210 | -0.407* |
|  | significance |  |  | <0.001 | <0.001 | 0.935 | 0.324 | 0.048 |
| **Cond.** | ρ |  |  | 1.000 | 0.791** | -0.374 | -0.579** | -0.355 |
| **[µS cm^-1^]** | significance |  |  |  | <0.001 | 0.095 | 0.003 | 0.088 |
| **pH** | ρ |  |  |  | 1.000 | -0.162 | -0.378 | -0.553** |
|  | significance |  |  |  |  | 0.482 | 0.069 | 0.005 |
| **DNA** | ρ |  |  |  |  | 1.000 | 0.716** | 0.459* |
| **[ng µL^-1^]** | significance |  |  |  |  |  | <0.001 | 0.021 |
| **AllBac** | ρ |  |  |  |  |  | 1.000 | 0.522** |
|  | significance |  |  |  |  |  |  | 0.004 |

**Supplementary Data Table S4:** Spearman rank order correlation results for samples prepared from lakebed sediment and lake water (test series II). **: The correlation is significant at the 0.01 level, n = 11.

|  |  | DNA | AllBac |
| --- | --- | --- | --- |
| TSS | ρ | -0.916^**^ | -0.964^**^ |
|  | significance | <0.001 | <0.001 |
| DNA | ρ | 1.000 | 0.967^**^ |
|  | significance |  | <0.001 |

**Supplementary Data Table S5:** Spearman rank order correlation results for the DNA extraction protocol modification using salmon sperm DNA as adsorption site blocker. *: The correlation is significant at the 0.05 level, **: The correlation is significant at the 0.01 level, n = 21.

|  |  | **TSS** | **DNA conc.** | **AllBac** | **DeTaCS** |
| --- | --- | --- | --- | --- | --- |
| **Salmon sperm DNA** | ρ | 0.000 | 0.712** | 0.379 | 0.515* |
|  | significance | 1.000 | <0.001 | 0.121 | 0.017 |
| **TSS** | ρ | 1.000 | -0.270 | 0.708** | -0.616** |
|  | significance |  | 0.237 | 0.001 | 0.003 |
| **DNA conc.** | ρ |  | 1.000 | 0.220 | 0.732** |
|  | significance |  |  | 0.381 | <0.001 |
| **AllBac** | ρ |  |  | 1.000 | -0.228 |
|  | significance |  |  |  | 0.363 |

**References**

Boyd, D., Weiss, D. S., Chen, J. C., and Beckwith, J. (2000). Towards single-copy gene expression systems making gene cloning physiologically relevant: lambda InCh, a simple Escherichia coli plasmid-chromosome shuttle system. *J. Bacteriol*. 182, 3, 842-847. doi: 10.1128/JB.182.3.842-847.2000

Cooper, S., and Helmstetter, C. E. (1968). Chromosome replication and the division cycle of *Escherichia coli* Br. *J. Mol. Biol.*, 31, 3, 519-540. doi: 10.1016/0022-2836(68)90425-7

Reischer, G. H., Kasper, D. C., Steinborn, R., *et al.* (2006). Quantitative PCR method for sensitive detection of ruminant fecal pollution in freshwater and evaluation of this method in alpine karstic regions. *Appl. Environ. Microbiol.* 72, 8, 5610-5614. doi: 10.1128/AEM.00364-06
